# A review of filarial nematodes parasitizing tick vectors: unraveling global patterns in species diversity, host associations, and interactions with tick-borne pathogens

**DOI:** 10.1186/s13071-025-06690-6

**Published:** 2025-02-12

**Authors:** Oluwaseun D. Ajileye, Guilherme G. Verocai, Jessica E. Light

**Affiliations:** 1 Ecology and Evolutionary Biology Program, Texas A&M University, College Station, TX USA; 2 Department of Ecology and Conservation Biology, Texas A&M University, College Station, TX USA; 3 Department of Veterinary Pathobiology, College of Veterinary Medicine and Biomedical Sciences, Texas A&M University, College Station, TX USA

**Keywords:** *Acanthocheilonema*, Biological vectors, *Cercopithifilaria*, Coinfections, Filarioidea, Ixodida, *Monanema*, North America, Onchocercidae, Tick microbiome

## Abstract

Ticks are well-known vectors of pathogens, including filarial nematodes, which can affect animal and human health. This review synthesizes current knowledge of filarial nematodes that utilize ticks as vectors, focusing on the Onchocercidae. Five genera of onchocercid filarial nematodes (*Cercopithifilaria*, *Cherylia*, *Cruorifilaria*, *Monanema*, and *Yatesia*) have demonstrated vector–parasite relationships with ticks, while *Acanthocheilonema* has only demonstrated molecular detection without confirmed vector competence. Vector competence has been experimentally proven for several species through documented development to infective stages. However, our understanding of tick-borne filarial nematodes remains limited owing to insufficient detection efforts and methodological challenges. Detection methods include traditional dissection, advanced imaging techniques such as microcomputed tomography (micro-CT), and molecular approaches, each with distinct advantages and limitations. To address knowledge gaps, future research should focus on conducting large-scale surveys of tick populations and wildlife to investigate vector competence, health impacts, and coinfection dynamics. Multi-disciplinary collaboration and community involvement in tick surveillance will advance our understanding of tick-borne filarial nematode ecology and epidemiology. Future research priorities include confirming vector competence where only molecular detection exists, investigating natural transmission patterns, and understanding the mechanisms influencing successful development in tick vectors. By addressing these knowledge gaps through systematic surveillance and experimental studies, we can better understand these complex host–parasite–vector relationships and their implications for animal health.

**Graphical Abstract:**

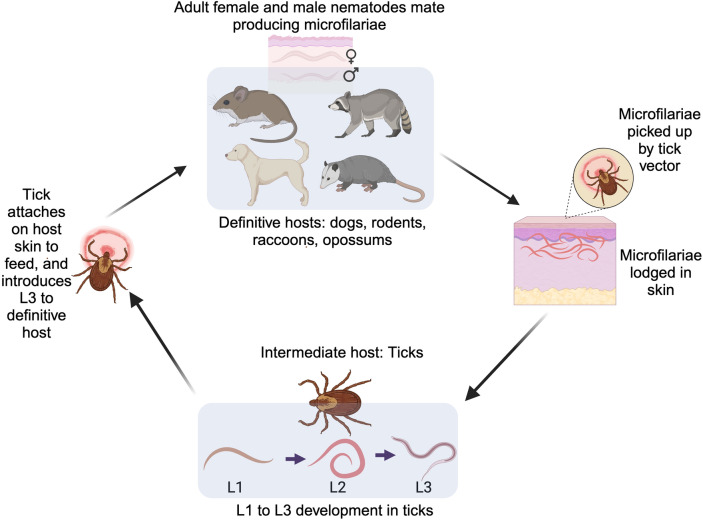

## Background

Ticks (Ixodida) are blood-feeding arthropods known to spread (i.e., vector) a range of pathogens of medical and veterinary importance [1, 2]. Ticks transmit a variety of disease-causing microbial taxa, spanning bacteria eliciting Lyme borreliosis, anaplasmosis, rickettsiosis, and ehrlichiosis [3–5]; viruses causing Powassan, Heartland, and tick-borne encephalitis infections [3, 6–8]; *Babesia* and *Hepatozoon* protozoan parasites causing babesiosis and hepatozoonosis, respectively [9–11]; and macroparasites, including filarial nematodes of medical and veterinary importance [12–14]. Filarial nematodes belonging to the superfamily Filarioidea Weinland, 1858 are further divided into two families: Filariidae Cobbold, 1864 and Onchocercidae Leiper, 1911. These nematodes have indirect life cycles, requiring arthropod intermediate hosts (biological vectors) to complete their development and transmission. Despite the diverse arthropod vectors utilized by filarial nematodes, only a few Onchocercidae species have been shown to use ticks as primary intermediate hosts [15]. Most Filarioidea rely on blood-sucking insects, such as mosquitoes, fleas, black flies, and biting midges, as primary vectors [16, 17]. Tick-transmitted filarioid nematodes show specific host associations; for example, *Cercopithifilaria bainae* infects domestic dogs and *Monanema marmotae* parasitizes groundhogs (*Marmota monax*) [12, 18]. Similarly, other arthropod-transmitted filariae demonstrate host specificity, including some filarioid species that are agents of neglected tropical diseases in humans, such as the black fly-transmitted *Onchocerca volvulus* and the mosquito-transmitted *Wuchereria bancrofti* in humans [19, 20].

Morphologically, filarial nematodes are elongated and threadlike, with long cylindrical bodies coated in a rigid cuticle with oral and anal openings, sensory papillae, and reproductive organs [16, 21, 22]. Nematode sexes are separated, with males and females sometimes being morphologically different; males may be shorter with coiled posterior ends, whereas females tend to be more elongated. Microfilariae are the initial larval stages of development of filarial nematodes, which develop in arthropod vectors into first-stage larva (L1), second-stage larvae (L2), and subsequently to third-stage larvae (L3), which is infective to the definitive host [16]. Vertebrate species typically serve as the definitive host for filarial nematodes, wherein adult nematodes attain sexual maturity and reproduce, with females producing fertilized eggs that hatch into microfilariae. Microfilariae can circulate throughout the vertebrate host body and are often ingested by arthropod vectors such as insects (Insecta) and acarines (Acari; ticks and mites) during blood-feeding activities, where the definitive host enables parasite reproduction and continued transmission [22–24].

The potential role of tick vectors in propagating filarial nematodes and affecting animal and human health remains understudied. Several studies have examined and identified filarial nematodes in both hard (Ixodidae) and soft (Argasidae) ticks [14, 25–35], thereby providing evidence that ticks can indeed vector filarial nematodes. Tick feeding strategies can significantly contribute to the biological transmission of filarial nematodes in several ways, including the duration of attachment, feeding preferences, and developmental stages. While slow-feeding ixodid ticks can remain attached to their hosts for several days or weeks, with some species feeding for up to 2 weeks [1], experimental studies by Brianti et al. [36] showed that extended feeding duration alone does not determine successful filarial nematode transmission. The typical development from microfilariae to infective L3 stage often takes longer than a single tick feeding period, usually around 30 days [36]. While this extended development time increases the likelihood of successful transmission to a new vertebrate host during subsequent feeding, reinfection of the same host during a single blood meal is unlikely. In addition, feeding patterns and host associations of ticks can play a crucial role in the life cycle of filarial nematodes [37]. For example, Ramos et al. [38] showed that *Cercopithifilaria rugosicauda* utilizes *Ixodes ricinus* nymphs to parasitize roe deer (*Capreolus capreolus*) in southern Italy. The timing of nymphal activity coincides with host availability, demonstrating how filarial transmission occurs through monotropic tick species—ticks that primarily parasitize specific host species.

While tick-borne filarial nematodes have been documented in various animal hosts, their impact on animal health remains poorly understood. Currently, there are no documented cases of tick-borne filarial nematodes affecting livestock production, and their economic significance, if any, requires further investigation. In companion animals, *Cercopithifilaria* spp. can infect dogs, with some naturally infected individuals developing dermal nodules, though these infections are rarely associated with clinical signs [23, 32, 36]. While treatments such as topical medications and corticosteroids are available, much remains to be understood about the pathogenicity and epidemiology of these infections [39].

In this review, we aim to synthesize and provide a comprehensive overview of the current knowledge on the global diversity of filarial nematode species that use ticks as vectors. Among the family Onchocercidae, development and transmission require arthropod intermediate hosts, with several genera specifically adapted to using ticks as vectors. Therefore, we focus on genera within Onchocercidae, reporting their known tick vectors and other arthropod vectors. In addition, using North America as a case study, we highlight significant gaps in the distribution and prevalence of tick-borne filarial nematodes and explore potential interactions between filarial nematodes and tick-borne pathogens and discuss their implications for infection dynamics and host immune responses. This review will cover the strengths and weaknesses of existing and current methods to detect filarial nematodes in tick vectors, emphasizing the need for targeted, large-scale research efforts focusing on known tick hotspots and risk interfaces. By synthesizing available information and critically assessing published literature, we aim to understand tick-borne filarial transmission among domestic and wildlife animals in natural ecosystems, with a focus on identifying knowledge gaps regarding these nematodes in North America to guide future research directions.

### Roles of vertebrate hosts and tick vectors in the transmission of filarial nematodes

**Fig. 1:**
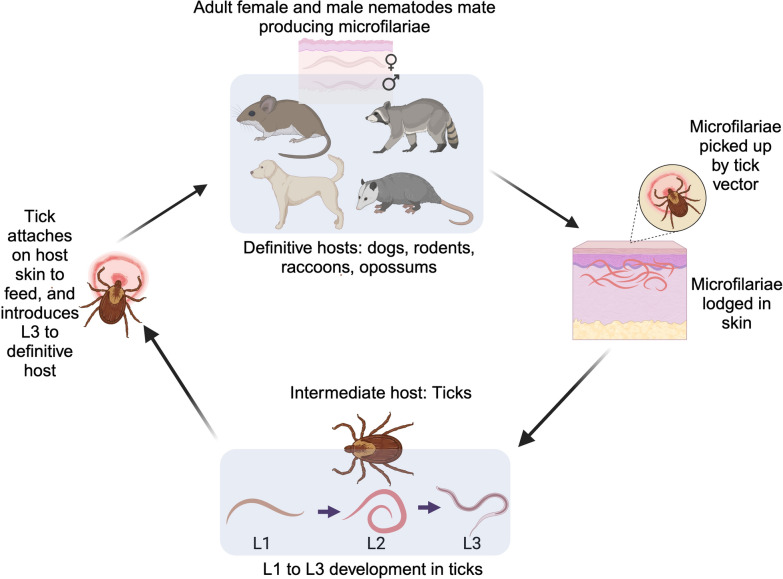
Transmission cycle of filarial nematodes in tick vectors and vertebrate hosts. The cycle begins when a tick vector introduces L3 larvae into the definitive host (e.g., dogs, rodents, raccoons, and opossums) through skin bites. Within the host, L3 larvae develop into adult nematodes that mate and produce microfilariae, which circulate in the skin or blood of the host. When a new tick feeds on an infected host, it ingests the microfilariae, which then develop into infective L1 to L3 larvae within the tick, completing the transmission cycle. Illustration created on Biorender.com

All vertebrate classes except fish serve as definitive hosts for filarial nematodes, enabling their development and transmission [40]. Vertebrate hosts harboring filarial nematodes allow the parasite to complete their life cycle, thus facilitating their transmission to tick vectors (Fig. 1). For instance, the life cycle of *C. rugosicauda* illustrates the synchronized timing between tick vectors and vertebrate hosts, which is essential for successful transmission. Development in *I. ricinus* takes 8 weeks for microfilariae to reach the infective L3 stage, followed by 24 weeks in roe deer for L3 to mature into adults [30, 38]. Adult nematodes produce microfilariae that concentrate in dermal tissues, coinciding with tick feeding periods and enabling continued transmission.

The efficiency of filarial nematode transmission between vertebrate hosts and tick vectors may depend on host–vector compatibility. The availability of compatible hosts and vector feeding preferences may determine the geographic distribution of vector-borne helminths. For instance, domestic dogs serve as efficient reservoirs for filarial nematodes by simultaneously maintaining long-term infections with multiple species and their ability to sustain infections, which makes them consistent sources of microfilariae for brown dog ticks throughout the vector active season [14, 36].

### Complexities of tick-borne filarial nematode transmission and development in vertebrate hosts

Early studies by Bain [28], Bain et al. [35], and Beaver and Burgdorfer [29] showed that the hindgut, hemocoel, fat cells, and salivary gland ducts of ticks provide a favorable environment for the development of certain filarial nematodes. In these cases, ticks ingest microfilariae when feeding on an infected vertebrate host [28] (Fig. 1). These microfilariae then migrate to specific tick tissues to find suitable conditions for growth and transformation into infective larvae. For example, many microfilariae migrate from the tick midgut to the hemocoel (i.e., body cavity), where they molt into L1, L2, and subsequently into infective L3, a process that may take 30 days [36]. L3 larvae have been documented in tick salivary glands [13, 36]; however, their transmission to vertebrate hosts via salivary glands is yet to be established. As ticks feed for several days, they regurgitate saliva into the bite site, potentially countering host hemostasis and inflammatory responses [22, 41–43]. Tick feeding can potentially deliver L3 larvae into the dermal skin layers of the host, setting off local reactions and enabling nematode transfer [18] (Fig. 1).

The concentration of microfilariae in the skin of infected vertebrates near common tick attachment sites (such as the ears) implies a specific attraction of filarial nematodes to chemical substances in tick saliva, as demonstrated by Ko [18]; a similar mechanism was proposed by Moorhouse [43]. If broadly applicable, this attraction could enhance the probability of microfilariae being ingested by the tick vector during blood feeding, facilitating their transmission to the vector and subsequently to new susceptible vertebrate hosts (Fig. 1). The specific vertebrate tissue harboring these transmitted microfilariae (e.g., skin or blood) corresponds to the feeding strategies of the arthropod vector on hosts [44]. For instance, microfilariae inhabiting subdermal skin layers may be ingested by ticks during their blood-feeding process. Alternatively, some circulating blood-borne microfilariae can be consumed through vessel lacerations by arthropod vectors such as mosquitoes and other flies that feed on blood using anticoagulant strategies. Thus, vertebrate microfilariae niche accessibility aligns with vector hematophagous behavior, which propagates initial establishment within intermediate hosts [45].

The life cycles of filarial nematodes within arthropod vectors are initiated when blood-feeding uptake introduces vertebrate-derived microfilariae into the vector system. The complex process by which nematodes develop from microfilariae to L3 within ticks involves multiple stages that occur in specific organs and tissues, with translocation through the stomach walls [22] (Fig. 1). While the role of saliva in tick-feeding has been well documented in tick-borne pathogens [46, 47], knowledge gaps exist on how filarial nematodes interact with and potentially impact tick organs. As microfilariae and developing larvae migrate through various tick tissues—from gut epithelium to hemocoel and salivary glands—they may affect tissue integrity and function. The efficiency of L3 from tick-to-host transfer could depend on these tissue interactions; aspects of this process remain elusive and require further investigation to comprehend the mechanisms at play.

Once in the definitive host, the infective L3 typically molts and migrates. Some L3 may move from the skin via lymphatic vessels to other vertebrate host tissues to continue development. Others may remain in the skin to mature into adults that mate and produce microfilariae, enabling acquisition by new ticks to perpetuate further transmission cycles [32] (Fig. 1). The immunogenic nature of incoming L3 larvae may influence the establishment of infection in the vertebrate host and host immune defenses at bite sites or downstream migration routes can impede nematode survival. Deciphering the fundamental processes governing these multifaceted vertebrate–tick–parasite associations may necessitate a deeper understanding of the complex interaction and impact on the circulation of filarial nematodes, as this affects host health.

### Detection methods for tick-borne filarial nematodes

Detecting and screening tick vectors for filarial nematode infections is essential for assessing the prevalence of these nematodes in tick populations, identifying potential risk areas for transmission, and developing targeted control strategies [14, 36]. Various techniques, including dissection [36, 38], histopathological analyses [14], and molecular approaches, such as polymerase chain reaction (PCR) and DNA sequencing [12, 33] (Table 1), can be used to assess the status of tick infection with filarial nematodes. These methods differ in sensitivity, specificity, and practicality, making it essential to consider the strengths and weaknesses of each approach when designing studies or surveillance programs.

**Table 1:** Comprehensive assessment of methodologies employed in the detection of filarial nematodes in tick vectors

Approach	Strengths	Weaknesses
	Allows for the direct observation and morphological characterization of filarial nematodes	Labor-intensive and time-consuming, potentially missing less developed or smaller larval stages of filarial nematodes
	Provides information on the anatomical location and distribution of filarial nematodes within tick tissues	Requires skilled personnel to dissect ticks and identify filarial nematodes
Dissection and microscopic examination	Enables the collection of live filarial specimens for further studies	Limited throughput is not suitable for screening large numbers of ticks
	Facilitates the study of host–parasite interactions at the tissue level	Destructive to the tick samples and potential for mechanical damage to filarial nematodes during dissection
	Allows for the isolation and culture of filarial nematodes for downstream applications	Difficulty in preserving the integrity of delicate filarial structures
	Allows visualization of filarial nematodes within tick tissues	Limited resolution may hinder the identification of early developmental stages or small filarial species
	Provides information on the anatomical location and distribution of filarial nematodes	Some imaging techniques require specialized imaging equipment and expertise with limited penetration depth
Imaging techniques (X-ray micro-CT)	Enables the observation of different developmental stages and allows for assessing pathological changes in tick tissues caused by filarial nematodes	Sample preparation can be time-consuming, expensive, and may not provide definitive species identification, as CT scanning costs can be high
	Facilitates the study of host–parasite interactions and the potential for in vivo imaging of filarial nematode dynamics	Difficulty in distinguishing between filarial nematodes and other structures with similar morphology
	High sensitivity and specificity in detecting filarial nematodes	Requires specialized equipment and expertise and can be expensive, especially for high-throughput sequencing methods
	Ability to identify and differentiate filarial species and strains	May not provide information on the anatomical location or developmental stage of the filarial nematodes within the tick
Molecular techniques	Suitable for high-throughput screening of tick samples; it can also detect early developmental stages of filarial nematodes	Potential for cross-contamination or false- positive results and there may be difficulty identifying species if there are not suitable reference sequences
	Enables the discovery of novel filarial species and allows for the quantification of filarial nematode intensity of infection in ticks	Destructive to the tick samples and filarial nematodes and DNA degradation in poorly preserved samples may affect results
	Facilitates the study of genetic diversity and population structure of filarial nematodes	Inability to distinguish between viable and nonviable filarial nematodes

### Classical approaches to detecting tick-borne filarial nematodes

**Fig. 2:**
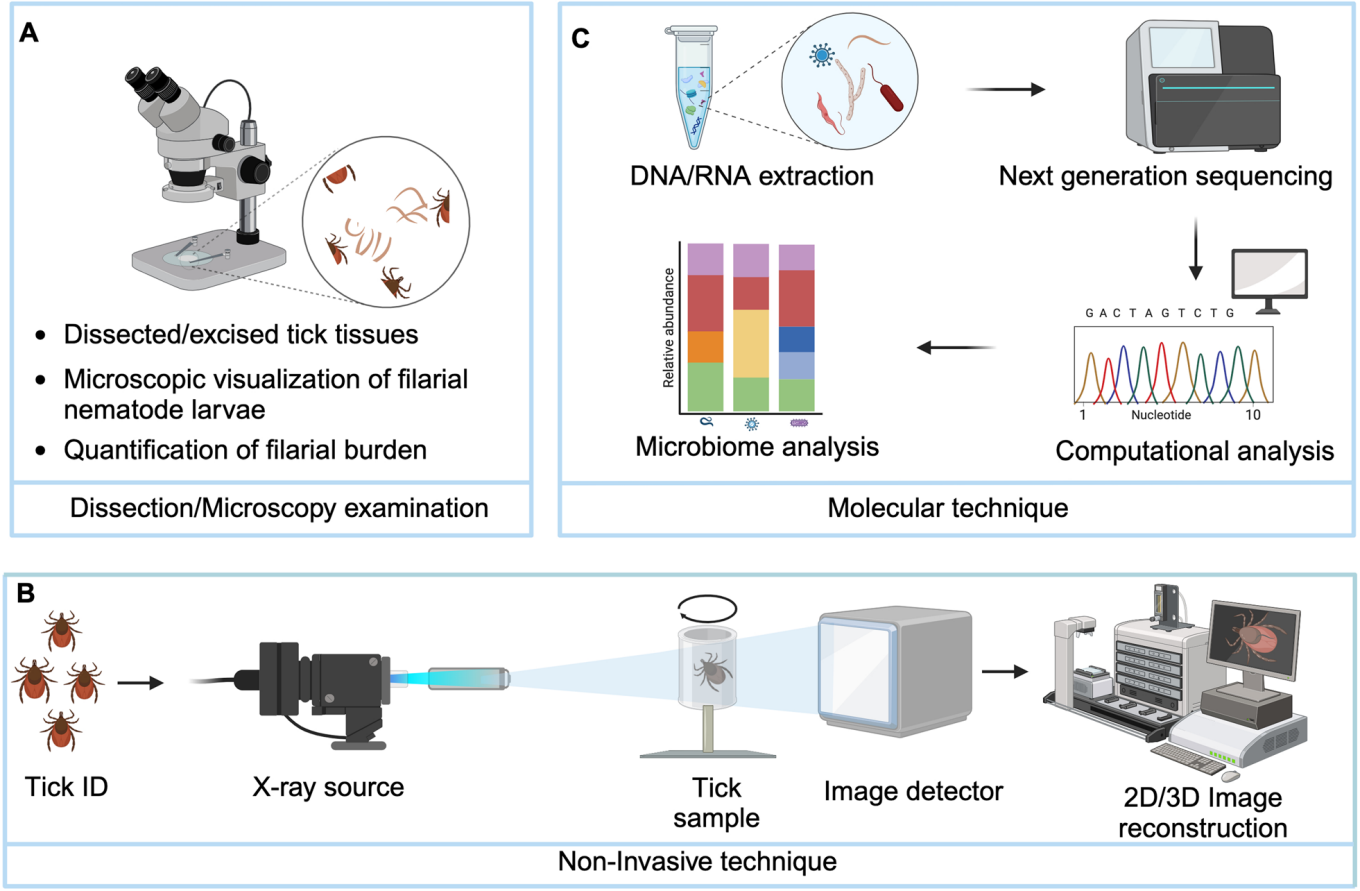
Complementary approaches for detecting and characterizing filarial nematodes in tick vectors. (**A**) Classical methods involve invasive techniques such as dissecting and microscopically examining excised tick tissues to visualize and quantify filarial nematodes; (**B**) noninvasive imaging techniques, such as X-ray micro-computed tomography (micro-CT), allow for the visualization and localization of filarial nematodes within tick samples without dissection, generating high-resolution two-dimensional (2D) and three-dimensional (3D) images; (**C**) molecular techniques, such as DNA/RNA extraction (often requiring the destruction of the sample), followed by PCR, sequencing, or next-generation sequencing and computational analysis, enable the identification and characterization of filarial nematodes at the nucleotide level. Illustration created on Biorender.com

Dissection and microscopic examination of ticks represent a classical means of investigating filarial infection and developmental progression within different tick tissues (Fig. 2A). Using these methodologies, filarial nematodes have been observed in hard tick species, including *Amblyomma cajennense* [31], *Haemaphysalis flava*, *H. japonica* [48], *Ixodes cookei* [18, 49], *I. scapularis* [29], and *I. ricinus* [30, 35, 38, 50]. Soft ticks, such as *Ornithodoros tartakovskyi* [51] and *O. talaje* [52], have also been found to carry filarial nematodes. Initial investigations into filarial nematode infections in ticks relied upon microscopic visualization methods for identification and morphological diagnosis. While microscopy and dissection techniques provided early verification that ticks harbor filarial nematodes, these methods have limitations in species identification. Morphological identification of filarial larvae is challenging owing to similar anatomical features among species and cryptic species complexes [53], particularly in early developmental stages. Therefore, while genus-level identification is possible through microscopy and dissection, molecular methods are typically needed for species-level determination.

Patton et al. [54] established comprehensive protocols for dissecting tick organs and tissues, and for the collection of biofluids (i.e., hemolymph, saliva), enabling examination of tick internal structures for the presence of pathogens. These methods, later applied by Santos et al. [55] to study filarial nematodes, can be adapted to target specific tissues where these parasites develop, facilitating visual identification of larvae within ticks. Studies have demonstrated the presence of filarial nematodes in tick organs, such as the hindgut, hemocoel, fat cells, and salivary ducts [18, 29, 35, 36]. Controlled infection experiments have directly validated the ability of specific tick genera, including *Amblyomma*, *Ixodes*, and *Rhipicephalus*, to effectively acquire and transmit filarial nematodes [18, 31, 36, 49, 56, 57]. Despite being constrained by morphological assessment limitations and operator expertise, adopting standardized dissection and preparation protocols would result in more consistent documentation of tissue distributions and quantification of tick-borne filarial burden. Thus far, no definitive reports have explicitly validated the precise anatomical locations occupied by developing L1–L3 filarial nematodes inside tick vectors. Expanding confirmation of occupied tick niches through other methods, such as targeted imaging and molecular approaches, may enable tracking of filarial occupation across tick tissues, thereby elucidating vector–parasite interface dynamics and transmission.

### Imaging techniques for detecting tick-borne filarial nematodes

The emergence of advanced biological imaging techniques, such as 3D X-ray microcomputed tomography (micro-CT), has ushered in exciting new potential for visualizing filarial nematode stages within arthropod vectors. Micro-CT instruments use X-ray beams to irradiate targeted samples; detectors capture unabsorbed photon signals to create complex 3D renderings that specialized software programs can analyze [58, 59] (Fig. 2B). This approach facilitates the examination of filarial nematodes inside arthropod vectors with minimal physical disruption of tissues. While some sample preparation involves fixation or contrast enhancement through staining, these processes are generally less invasive than traditional dissection methods.

The utilization of 3D X-ray micro-CT, supported by histopathological analysis, has facilitated the observation of *Acanthocheilonema spirocauda* larvae within the fat bodies (L1) and hemocoels (L3) of seal lice (Anoplura: *Echinophthirius horridus*) [58, 60]. *Acanthocheilonema spirocauda* is a filarial nematode that primarily infects seals and sea lions as definitive hosts. Hall et al. [61] used micro-CT imaging to visualize *Onchocerca* L1 larvae embedded within blackfly thoracic musculature. Previous studies demonstrated the potential of micro-CT imaging to reveal the precise spatiotemporal details of infection progression within arthropod vectors [58, 60, 61]. Application of micro-CT could help reveal favorable habitats, migratory routes, and life cycle transitions of larval stages of filarial nematodes in tick vectors.

### Molecular techniques for detecting tick-borne filarial nematodes

Cutting-edge molecular techniques have enabled more precise identification of nematode species and isolates transmitted by tick vectors globally (Table 1) (Fig. 2C). For example, genetic analyses have confirmed filarial nematodes in ticks such as *I. ricinus* [38] and *R. sanguineus* sensu lato (s.l.) [62–64]. These investigations employed molecular techniques, including DNA extraction, PCR, sequencing, and in some cases, alternative genetic assays such as metabarcoding, to identify filarial nematodes in ticks (Table 1). Molecular identification of filarial nematodes in tick vectors often relies on genetic markers such as 12S mitochondrial ribosomal RNA and cytochrome *c* oxidase subunit I [12, 24, 33, 34, 65]. When proper reference material is available, these markers allow accurate identification at both genus and species levels, particularly for distinguishing closely related species.

Employing PCR-based assays targeting specific filarial genes consistently facilitated the identification of prominent Onchocercidae species, such as those in the genera *Cercopithifilaria*, *Monanema*, and *Acanthocheilonema*, across numerous hard tick species (Table 2) [13, 62, 65–67]. These genetic screening techniques have enabled extensive detection of tick-borne filarial nematodes across tick vector populations, revealing the presence of pathogenic filariae spanning multiple tick genera and species. In North America, genetic analyses have enabled the discovery of filarial nematode DNA in several tick species. However, it is important to note that these molecular techniques cannot detect viable parasites or their developmental stages. There is a risk of detecting remnant DNA or recently ingested microfilariae from blood meals rather than active infections. Therefore, to confirm the biological relevance of these findings and avoid spurious detections, molecular assays should be combined with other methods, such as microscopy, 3D X-ray micro-CT, or experimental transmission studies. For example, molecular screening and high throughput sequencing (HTS) have facilitated filarial detection and species identification in *I. scapularis*, substantiated through multiple reports [3, 4, 13, 66]. Similar findings confirming the presence of filarial nematodes using comparable molecular techniques were also obtained in *A. americanum* ticks through studies by Zhang et al. [25] and Henning et al. [67] and in *R. sanguineus* s.l. by Lineberry et al. [33, 34].

**Table 2:** Global report of filarioid species recovered from tick vectors and their potential hosts

Geographic origin	Filarial species	Tick vectors	Vertebrate hosts/sampling	References
North America				
USA	*Acanthocheilonema* sp.	*Ixodes scapularis*	Tick dragging	[13]
USA	*Acanthocheilonema *spp.	*Amblyomma americanum*	Tick dragging	[25]
USA	*Cercopithifilaria bainae*	*Rhipicephalus sanguineus* s.l.	Dogs	[33, 34]
Canada	*Monanema marmotae*	*Ixodes cookei*	Rodents^a^	[18]
USA	*Monanema* sp.	*Amblyomma americanum*	Tick dragging	[67]
USA	*Monanema* sp.	*Ixodes scapularis*	Tick dragging	[66]
Africa				
South Africa	*Cercopithifilaria* spp.	*Rhipicephalus sanguineus* s.l.	Dogs	[63]
Kenya	*Monanema globulosa*	*Haemaphysalis leachi*	Rodents^b^	[84]
Asia				
Malaysia	*Cercopithifilaria bainae*	*Rhipicephalus sanguineus* s.l.	Dogs	[63]
Pakistan	*Cercopithifilaria grassi*	*Rhipicephalus* sp.	Dogs	[63]
Indonesia	*Cercopithifilaria bainae*	*Rhipicephalus sanguineus* s.l.	Cats	[12]
Philippines	*Cercopithifilaria bainae, Cercopithifilaria grassi*	*Rhipicephalus sanguineus* s.l.	Cats	[12]
India	*Cercopithifilaria bainae*, *Cercopithifilaria grassi*	*Rhipicephalus haemaphysaloides*	Dogs	[12]
China	*Cercopithifilaria bainae*	*Rhipicephalus sanguineus* s.l.	Dogs	[12]
Taiwan	*Cercopithifilaria bainae*	*Rhipicephalus sanguineus* s.l.	Dogs	[12]
Vietnam	*Cercopithifilaria bainae*	*Rhipicephalus sanguineus* s.l.	Dogs	[12]
Europe				
Romania	*Cercopithifilaria bainae*	*Dermacentor reticulatus*	Dogs	[102]
Greece	*Cercopithifilaria bainae*	*Rhipicephalus sanguineus* s.l.	Dogs	[103]
Greece, Italy, Spain	*Cercopithifilaria* sp.	*Rhipicephalus sanguineus* s.l.	Dogs	[14]
Italy	*Cercopithifilaria* sp.	*Rhipicephalus sanguineus* s.l.	Dogs	[36]
Greece, Italy, Spain, Portugal	*Cercopithifilaria* spp.	*Rhipicephalus sanguineus* s.l.	Dogs	[63]
Switzerland	*Cercopithifilaria grassii*	*Rhipicephalus sanguineus* s.l.	Dogs	[35]
Italy	*Cercopithifilaria grassii*	*Rhipicephalus sanguineus* s.l.	Dogs	[83]
Italy	*Cercopithifilaria rugosicauda*	*Ixodes ricinus*	Roe deer	[38]
Oceania				
Australia	*Cercopithifilaria johnstoni*	*Ixodes trichosuri*	Rats	[56]
Australia	*Cercopithifilaria* spp.	*Rhipicephalus sanguineus* s.l.	Dogs	[63]
South America				
Brazil	*Cercopithifilaria bainae*	*Rhipicephalus sanguineus* s.l.	Dogs	[55, 64, 94]
Brazil	*Cercopithifilaria* spp.	*Rhipicephalus sanguineus* s.l.	Dogs	[63, 94]
French Guiana	*Cercopithifilaria* sp.	*Amblyomma oblongoguttatum*, *Rhipicephalus sanguineus* s.l.	Opossums, tick-dragging	[24]
French Guiana	*Cruorifilaria* sp.	*Amblyomma cajennense*, *Amblyomma romitii*	Opossums, tick-dragging	[24]
Experimental studies				
Iran	*Acanthocheilonema viteae*	*Ornithodoros tartakovskyi*	Rodents^c^	[51]
Unknown location	*Acanthocheilonema viteae*	*Ornithodoros tartakovskyi*	Rodents	[86]
USA	*Acanthocheilonema viteae*	*Ornithodoros tartakovskyi*	Rodents	[27, 85]
Spain	*Acanthocheilonema dracunculoides*	*Rhipicephalus sanguineus* s.l.	Dogs	[26]
French Guiana	*Cherylia guyanensis*	*Ixodes ricinus*	Marsurpials^d^	[87]
France	*Monanema martini*	*Rhipicephalus sanguineus* s.l., *Rhipicephalus turanicus*,* Hyalomma truncatum*	Rodents^b^	[57]
Unknown location	*Yatesia hydrochoerus*	*Amblyomma cajennense*,* Amblyomma americanum*	Rodents^e^	[31]

For each filarial species, the following information is provided: geographic region of discovery, associated tick vector species, methodology used for tick collection (e.g., tick dragging or sampling from vegetation; collection from vertebrate hosts), and relevant literature references

The taxonomy of the *Rhipicephalus sanguineus* group has changed in recent years and the actual tick species reported in these studies may be unknown. For this reason, ticks are referred to as *R. sanguineus* s.l. in this table. For more details see: Dantas-Torres et al. [108]

^a^Groundhog

^b^Mouse

^c^Gerbil

^d^Opossum

^e^Capybara

In Europe, *C. rugosicauda* was initially detected in the hard tick *I. ricinus* through dissection and morphological studies across Austria, Germany, and France [30, 35, 50]. Later, Ramos et al. [38] used both morphological and molecular techniques, specifically dissection, microscopy, PCR, and sequencing, to detect *C. rugosicauda* L3 in *I. ricinus* nymphs from southern Italy, confirming the distribution of this nematode across Europe. Similarly, Binetruy and Duron [24] used molecular methods to detect filarial nematodes in ticks collected from French Guiana, South America. They discovered filarial species, including representatives from the genera *Cercopithifilaria*, * Cruorifilaria*, and putative *Dipetalonema*-related nematodes, inhabiting *Amblyomma*, *Ixodes*, and *Rhipicephalus* ticks in the region. Further characterization through multi-locus sequence analysis by Bruley and Duron [68] provided additional insights into tick-borne filarial diversity in this region.

### Filarial nematodes and tick microbiome interactions

There is growing interest in understanding how the tick microbiome, which refers to the diverse community of microorganisms residing within the tick, contributes to modifying tick life cycles as well as pathogen and parasite acquisition and transmission. Ticks harbor a diverse microbiome comprising endogenous and exogenous bacteria, fungi, protozoa, viruses, and filarial nematodes that can colonize tick organs [3, 55, 69]. Owing to their hematophagous lifestyle, host tissue salivation, and frequent host switching, ticks provide suitable habitats for various microbes [70]. Ticks are vectors of numerous medical and veterinary pathogens, including bacteria (such as Rickettsiales, Anaplasmataceae, and spirochetes), protozoa, and viruses, and individual ticks may be concurrently infected (coinfected) with multiple microbial pathogens and filarial nematode larvae [3, 69]. Despite the frequent occurrence of multiple infections, most investigations typically examined tick-borne microbes [71, 72] and filarial nematodes in ticks individually rather than as an integrated multi-kingdom community [14, 24, 33, 66].

**Fig. 3:**
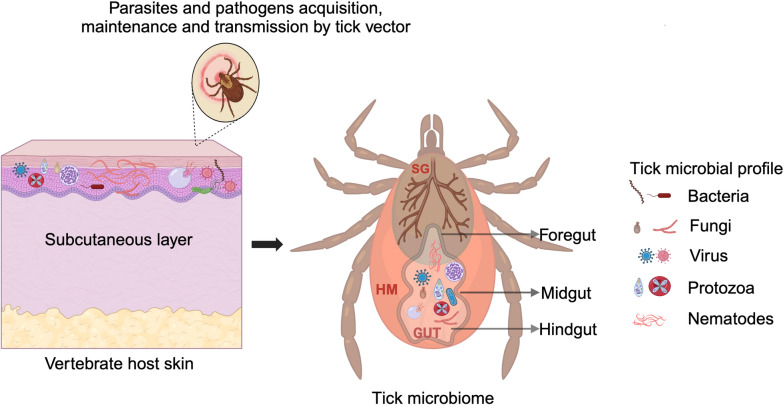
Potential interactions between tick microbiome and pathogen transmission across tick tissues. The tick microbiome, consisting of a diverse array of microorganisms acquired from the vertebrate host during blood feeding, colonizes different regions of the tick gut. These microbes can potentially influence pathogen acquisition, maintenance, and transmission through diverse biological interactions, ultimately shaping pathogen transmission dynamics from the tick vector to the vertebrate host. Understanding the complex interplay between the tick microbiome and pathogen transmission is essential for developing effective tick-borne disease control strategies. SG, salivary glands; HM, hemocoel. Illustration created on Biorender.com and adapted from [92]

Coinfection adds complexity to the tick microbiome, creating interactions that may be mutualistic, antagonistic, or manipulative between coexisting organisms [73] (Fig. 3). While studies in other arthropod vectors show that such interactions can affect transmission dynamics and host health [74, 75], relationships between tick-borne pathogens and filarial nematodes remain largely unexplored. One documented example is the association between filarial nematodes and endosymbiotic *Wolbachia* bacteria in tick vectors [3, 25]. Using high-throughput sequencing, Cross et al. [3] demonstrated a significant positive correlation between onchocercid filarial nematodes and *Wolbachia* in *I. scapularis* ticks from Wisconsin. Understanding these and similar relationships could provide insights into disease transmission patterns and tick physiology.

Studies have documented co-occurrence of filarial nematodes with other pathogens in tick vectors. For instance, Zhang et al. [26] found filarial nematodes alongside other pathogens in *A. americanum* ticks, while Ramos et al. [69] and Santos et al. [55] observed *C. bainae* co-occurring with *Hepatozoon canis* in *R. sanguineus* s.l. However, while these studies document co-occurrence, the potential interactions between these organisms and their influence on transmission dynamics and vector competence remain to be determined.

### Potential interactions between filarial nematodes and other tick-borne pathogens

The potential impact of filarial nematodes on tick-borne disease transmission remains poorly understood, despite the well-established role of ticks as vectors of numerous pathogens in humans and animals. This interplay between filarial nematodes and established tick-borne pathogen transmission systems presents an important area for further research. Ticks transmit a wide range of disease-causing agents, including *Borrelia burgdorferi*, which causes Lyme disease, *Rickettsia rickettsii*, which is responsible for Rocky Mountain spotted fever, and *Anaplasma* species, which leads to anaplasmosis [76, 77]. Key tick anatomical structures (e.g., guts, hemocoel, and salivary glands) serve as critical interfaces between the tick, its pathogens, and the vertebrate host (Fig. 3). These tissues provide distinct microenvironments potentially affecting pathogen and parasite establishment and development. While interactions between tick-borne pathogens and tick tissues have been well studied [78, 79], how filarial nematodes interact with these pathogens and tick tissues [22] requires further investigation to know the mechanism at play. These interactions likely vary with vertebrate host species, tick species, tick developmental stages and sex, and blood-feeding status (i.e., engorged versus nonengorged ticks).

As filarial nematodes migrate through tick tissues, they encounter various pathogens within the tick microbiome [80–82]. These interactions occur in different tick compartments, particularly in the hindgut, where many pathogens undergo critical developmental processes and transovarial transmission (i.e., the passage of pathogens from an adult female tick to her eggs). The presence of filarial nematodes during migration through these tissues may influence pathogen distribution and the local microbiome composition in ways that could affect transmission dynamics (Fig. 3). However, the specific mechanisms and consequences of these interactions remain largely unexplored.

### Tick vectors of filarial nematodes: global perspective, current evidence, and research needs

Members of the superfamily Filarioidea (families Filariidae and Onchocercidae) parasitize primarily terrestrial vertebrate species, including mammals, birds, reptiles, and amphibians. The Onchocercidae show broader vector associations than Filariidae [45], with several genera specifically adapted to tick transmission [16]. Our current understanding of tick-borne filarial diversity and distribution remains incomplete [13, 33, 67], with five genera (*Cercopithifilaria*, *Cherylia*, *Cruorifilaria*, *Monanema*, and *Yatesia*) documented as using ticks as vectors (Table 3). Some of these genera may also utilize other arthropod vectors, reflecting their adaptability to different transmission routes (Table 4).

**Table 3:** List of known tick-borne filarial nematode genera (family Onchocercidae), their vertebrate hosts, and geographic regions

Filarial species	Vertebrate hosts	Geographic regions	References
*Acanthocheilonema*	Dogs, cats, wild carnivorans	Africa, Asia, Europe, North America	[93]
*Cercopithifilaria*	Dogs, cats, wild carnivores, deer, rodents	Africa, Asia, Australia, Europe, South and North America	[32]
*Cherylia*	Marsupials	South America	[87]
*Cruorifilaria*	Domestic and wild carnivores	Europe, South America	[94–96]
*Monanema*	Rodents, other small mammals	Africa, Australia, North America	[57, 84, 97–100]
*Yatesia*	Capybaras	South America	[31, 94, 101]

**Table 4:** Filarial nematode genera with unknown or non-tick vectors listed by family and genus, vertebrate and intermediate hosts, and their geographic regions

Family	Genus	Vertebrate hosts	Intermediate hosts	Geographic region
Filariidae	*Bhalfilaria*	Birds: chicken; mammals: buffalo, cattle, sheep	Biting midges, blackflies	Asia, Europe, North America
	*Cardiofilaria*	Birds: stork, chicken	Mosquitoes, biting midges	Asia, Australia, Europe, South and North America
	*Filaria*	Mammals: carnivores, nonhuman primates, rodents, ungulates	Mosquitoes	Europe, Asia, Africa, South and North America
	*Indofilaria*	Mammals: carnivores, primates, ungulates	Biting midges, Mosquitoes	Africa, Asia
	*Loa*	Mammals: humans, primates	Deer flies	Central and West Africa
	*Onchocerca*	Mammals: human, large mammals	Blackflies	Worldwide
	*Parafilaria*	Mammals: cattle, horses, ungulates	Flies	Africa, Asia, Europe, North America
	*Parasaurositus*	Reptiles: non-avian reptiles	Unknown	South Asia
	*Pseudodiomedenema*	Birds	Unknown	India
	*Pseudofilaria*	Birds	Biting midges	Africa, Asia, Europe, North America
	*Scytofilaria*	Mammals: primates, rodents	Mosquitoes	Southeast Asia
	*Struthiofilaria*	Birds: ostrich	Biting midges	Africa, Asia, Europe
	*Suifilaria*	Mammals: domestic and wild boars	Mosquito	Asia, Europe
	*Tipasella*	Reptiles: non-avian reptiles	Potentially ticks	Africa
	*Tricheilonema*	Mammals: domestic and wildlife mammals	Vector not needed	Africa, Asia, Europe, and the Americas
Onchocercidae	*Brugia*	Mammals: cats, dogs, raccoons, humans	Mosquitoes	Asia
	*Breinlia*	Mammals: opossums, rats	Mosquitoes	Australia, Southeast Asia, India
	*Chandlerella*	Birds: grackles, blue jay	Biting midges	North America
	*Dirofilaria*	Mammals: bear, cats, dogs, raccoons, pigs, porcupines, wild boars	Mosquitoes	Worldwide
	*Elaeophora*	Mammals: cattle, horse, moose, red deer, sheep	Horseflies	Africa, Asia, Europe, and North America
	*Foleyella*	Reptiles: lizards, chameleons	Mosquitoes	North America
	*Litomosa*	Mammals: bats	Unknown	France
	*Litomosoides*	Mammals: bats, opossums, rodents	Mites	Europe and America
	*Mansonella*	Primates: humans, carnivores	Biting midges, blackflies	Africa, Central, South and North America
	*Ochoterenella*	Amphibians: toads	Unknown	South America
	*Onchocerca*	Mammals: carnivores, humans, ungulates	Blackflies, biting midges	Worldwide
	*Piratuba*	Reptiles: lizards	Unknown	South America
	*Sarconema*	Birds: swans	Lice	North America
	*Waltonella*	Amphibians: bullfrogs	Mosquitoes	Africa, Asia, Europe, South and North America
	*Wuchereria*	Mammals: humans	Mosquitoes	Africa, Asia, Caribbean Islands, South America

Vector competence has been experimentally shown for several filarial species through demonstrated development to infective stages (Table 5). *Cercopithifilaria bainae* and *C. grassii* develop successfully in *R. sanguineus* s.l., which serves as their primary vector worldwide [32, 35, 83], while *C. rugosicauda* shows specific adaptation to *I. ricinus* [30, 38]. Among *Monanema* species, confirmed vector relationships include *M. marmotae* in *I. cookei*, *M. globulosa* in *Haemaphysalis leachi*, and *M. martini* in multiple tick species (*R. sanguineus* s.l.,* R. turanicus*, and *Hyalomma truncatum*) [18, 57, 84]. *Acanthocheilonema viteae* represents the only species in its genus with confirmed development in tick vectors, specifically the *Ornithodoros* species [51, 85, 86], although *Acanthocheilonema* DNA has been detected in other tick species without confirmed vector competence. Experimental studies have also demonstrated *Yatesia hydrochoerus* development in *A. cajennense* complex ticks, and *C. guyanensis* shows laboratory development in *I. ricinus*, although the latter represents a non-natural vector relationship [31, 87].

**Table 5:** Filarial nematodes and their tick–vector associations: host relationships, evidence for vector competence, and relevant references

Filarial species	Tick vectors	Vertebrate hosts	Vector status; evidence (if known)	References
*Acanthocheilonema*				
*Acanthocheilonema dracunculoides*	*Rhipicephalus sanguineus* s.l. (suspected)	Dogs	Confirmed; experimental studies	[26, 104]
*A. viteae*	*Ornithodoros moubata*, *O. tartakovskyi*	Gerbils	Confirmed; experimental studies	[27, 51, 85, 105]
*Cercopithifilaria*				
*Cercopithifilaria bainae*	*R. sanguineus* s.l	Dogs	Confirmed	[36, 94, 106]
*C. grassi*	*R. sanguineus* s.l.	Dogs	Confirmed	[32, 106]
*C. rugosicauda*	*Ixodes ricinus*	Roe deer	Confirmed	[30, 35, 38, 50]
*Cherylia*				
*Cherylia guyanensis*	*I. ricinus*	Gray four-eyed opossum	Laboratory development only	[87]
*Cruorifilaria*				
*Cruorifilaria* sp.	*Amblyomma romitii*, *A. cajennense*	Capybara	Unconfirmed; DNA detected	[24]
*Monanema*				
*Monanema marmotae*	*I. cookei*	Groundhog	Confirmed	[18, 49]
*M. globulosa*	*Haemaphysalis leachi*	Striped grass mouse	Confirmed	[84]
*M. martini*	*R. sanguineus* s.l*.*, *R. turanicus*,* Hyalomma truncatum*	Nile rat	Confirmed	[57, 100]
*Yatesia*				
*Yatesia hydrochoerus*	*A. cajennense*,* A. americanum*	Capybara	Experimental development	[31]

These relationships likely reflect ecological adaptations, where tick feeding behaviors—including wide host range and prolonged attachment—facilitate filarial transmission. For instance, the widespread distribution of *Cercopithifilaria* species appears to be facilitated by the association with cosmopolitan tick vectors such as *R. sanguineus* s.l. [14]. Molecular detection methods have identified additional potential tick-filarial associations awaiting confirmation of vector competence, including *Cruorifilaria* DNA in *Amblyomma* species and *Monanema*-related sequences in various tick species [13, 24, 25, 66, 67]. Research priorities include confirming vector competence where only molecular detection exists and investigating natural transmission patterns in endemic areas. Studies should focus on documenting complete developmental cycles in tick vectors, identifying factors that influence successful transmission, and understanding the specificity of tick-filarial relationships. Standardized methods are needed to differentiate between transient presence of filarial DNA from blood meals and established infections in tick tissues.

### Potential factors limiting tick-borne transmission of major filarial nematodes

The transmission of filarial nematodes, particularly the well-studied genera *Brugia*, *Dirofilaria*, and *Onchocerca*, has been predominantly associated with insect vectors (Table 4). For example, *Onchocerca* species develop in black flies and biting midges [16], while *Dirofilaria* and *Brugia* species use mosquitoes as their primary vectors [88]. The apparent inability of ticks to transmit these nematodes raises intriguing questions about vector–parasite specificity and the evolutionary processes shaping these relationships. Some filarial nematodes may depend on their arthropod vectors extensively, leading to highly specialized interactions. While this dependency may provide plausible explanations for the absence of tick-borne transmission in some filarial nematodes, it is important to consider anatomical and physiological limitations. Dipterans possess distinct circulatory systems and specific tissues, such as Malpighian tubules, which are crucial for filarial larval development in some species [89]. In contrast, ticks have an open circulatory system and different organ arrangements [1]. The internal structure of ticks, lacking specific organs found in insects, may not provide the necessary anatomical environment for the development of some filarial nematodes. The feeding behavior of vectors is another critical factor. While mosquitoes and other blood-sucking insects typically feed quickly, aligning with the transmission strategy of many filarial nematodes, hard ticks feed slowly over several days [1]. This prolonged feeding pattern might be incompatible with the life cycle and transmission dynamics of some filarial species that have evolved to be transmitted during brief feeding events. The feeding duration mismatch could disrupt the delicate timing for successful filarial nematode transmission and establishment. Ticks possess distinct innate immune responses that differ from insects [90, 91]. However, the specific role of tick immunity in filarial nematode establishment and development remains to be investigated through experimental studies.

### Knowledge gaps of tick-borne filarial nematodes in North America

North America lacks thorough investigations of tick-borne filarial associations and transmission dynamics among endemic tick vectors and vertebrate hosts. The only known filarial nematodes documented in North America belong to the genera *Acanthocheilonema*, *Cercopithifilaria*, and *Monanema* (Table 2), with ixodid tick vectors implicated in transmitting these filarial nematodes to their definitive hosts. In contrast to findings from South America, Europe, Africa, Australia, and Asia, this limited evidence highlights substantial knowledge gaps in the biodiversity, host associations (both definitive and intermediate), and geographic distribution of tick-borne filarial nematodes across North America. Expanded surveillance of filarial nematodes in North American ticks is warranted to characterize the distribution and diversity of unknown tick-borne parasites and their potential tick vectors (Table 4).

## Conclusions

This review highlights our current understanding of tick-borne filarial nematodes and identifies critical knowledge gaps. Key priorities include determining the diversity and distribution of these parasites through systematic surveillance, investigating vector competence and transmission dynamics, and understanding how coinfections with other tick-borne pathogens affect disease ecology. Research initiatives should focus on comprehensive surveys using modern detection methods, conducting experimental transmission studies, and investigating host–parasite–vector relationships. Through collaborative research efforts across disciplines, we can better understand these complex relationships and develop effective control strategies.

## Data Availability

No datasets were generated or analyzed during the current study.

## Abbreviations

PCR: Polymerase chain reaction
DNA: Deoxyribonucleic Acid
RNA: Ribonucleic Acid
rRNA: Ribosomal RNA
HTS: High throughput sequencing
L1, L2, L3: Larval stages 1, 2, and 3
CT or micro-CT: Microcomputed tomography
2D: Two-dimensional
3D: Three-dimensional
s.l.: Sensu lato (in the broad sense)
sp.: Species (singular)
spp.: Species (plural)

## References

[1] Sonenshine DE, Roe RM. Biology of ticks (second edition). 2014. Biological Sciences Faculty Books.1. https://digitalcommons.odu.edu/biology_books/1.

[2] de la Fuente J, Estrada-Pena A, Rafael M, Almazan C, Bermudez S, Abdelbaset AE, et al. Perception of ticks and tick-borne diseases worldwide. Pathogens. 2023;12:1258. 37887774 10.3390/pathogens12101258 PMC10610181

[3] Cross ST, Kapuscinski ML, Perino J, Maertens BL, Weger-Lucarelli J, Ebel GD, et al. Co-infection patterns in individual *Ixodes scapularis* ticks reveal associations between viral. Eukaryot Bacterial Microorgan Viruses. 2018;10:388. 10.3390/v10070388 PMC6071216 30037148

[4] Tokarz R, Tagliafierro T, Sameroff S, Cucura DM, Oleynik A, Che X, et al. Microbiome analysis of *Ixodes scapularis* ticks from New York and Connecticut. Ticks Tick Borne Dis. 2019;10:894–900. 31023629 10.1016/j.ttbdis.2019.04.011

[5] Narasimhan S, Rajeevan N, Liu L, Zhao YO, Heisig J, Pan J, et al. Gut microbiota of the tick vector *Ixodes**scapularis* modulate colonization of the Lyme disease spirochete. Cell Host Microbe. 2014;15:58–71. 24439898 10.1016/j.chom.2013.12.001 PMC3905459

[6] Shah T, Li Q, Wang B, Baloch Z, Xia X. Geographical distribution and pathogenesis of ticks and tick-borne viral diseases. Front Microbiol. 2023;14:1185829. 37293222 10.3389/fmicb.2023.1185829 PMC10244671

[7] Zhou H, Xu L, Shi W. The human-infection potential of emerging tick-borne viruses is a global public health concern. Nat Rev Microbiol. 2023;21:215–7. 36526809 10.1038/s41579-022-00845-3

[8] Tokarz R, Sameroff S, Tagliafierro T, Jain K, Williams SH, Cucura DM, et al. Identification of novel viruses in *Amblyomma**americanum*, *Dermacentor**variabilis*, and *Ixodes**scapularis* ticks. MSphere. 2018;3:e00614-e617. 29564401 10.1128/mSphere.00614-17 PMC5853492

[9] Swanson SJ, Neitzel D, Reed KD, Belongia EA. Coinfections acquired from *Ixodes* ticks. Clin Microbiol Rev. 2006;19:708–27. 17041141 10.1128/CMR.00011-06 PMC1592693

[10] Tokarz R, Jain K, Bennett A, Briese T, Lipkin WI. Assessment of polymicrobial infections in ticks in New York State. Vector-Borne and Zoonotic Dis. 2010;10:217–21. 10.1089/vbz.2009.0036 PMC2883481 19725770

[11] Piesman J, Mather TN, Sinsky RJ, Spielman A. Duration of tick attachment and *Borrelia burgdorferi* transmission. J Clin Microbiol. 1987;25:557–8. 3571459 10.1128/jcm.25.3.557-558.1987 PMC265989

[12] Bezerra-Santos MA, de Macedo LO, Nguyen VL, Manoj RR, Laidoudi Y, Latrofa MS, et al. *Cercopithifilaria* spp. in ticks of companion animals from Asia: new putative hosts and vectors. Ticks Tick Borne Dis. 2022;13:101957. 35504199 10.1016/j.ttbdis.2022.101957

[13] Namrata P, Miller J, Shilpa M, Reddy P, Bandoski C, Rossi M, et al. Filarial nematode infection in *Ixodes**scapularis* ticks collected from southern Connecticut. Vet Sci. 2014;1:5–15.

[14] Otranto D, Brianti E, Latrofa M, Annoscia G, Weigl S, Lia RP, et al. On a *Cercopithifilaria* sp. transmitted by *Rhipicephalus**sanguineus*: a neglected, but widespread filarioid of dogs. Parasit Vectors. 2012;5:1. 22212459 10.1186/1756-3305-5-1 PMC3259067

[15] Lefoulon E, Bain O, Bourret J, Junker K, Guerrero R, Cañizales I, et al. Shaking the tree: multi-locus sequence typing usurps current onchocercid (filarial nematode) phylogeny. PLoS Negl Trop Dis. 2015;9:e0004233. 26588229 10.1371/journal.pntd.0004233 PMC4654488

[16] Anderson RC. Nematode parasites of vertebrates. Wallingford: CABI Publishing; 2000.

[17] Wilson AJ, Morgan ER, Booth M, Norman R, Perkins SE, Hauffe HC, et al. What is a vector? Philos Trans R Soc B: Biol Sci. 2017;372:20160085. 10.1098/rstb.2016.0085 PMC5352812 28289253

[18] Ko RC. The transmission of *Ackertia**marmotae* Webster, 1967 (Nematoda: Onchocercidae) of groundhogs (*Marmota**monax*) by *Ixodes**cookei*. Can J Zool. 1972;50:437–50. 5022070 10.1139/z72-062

[19] Cháves-González LE, Morales-Calvo F, Mora J, Solano-Barquero A, Verocai GG, Rojas A. What lies behind the curtain: cryptic diversity in helminth parasites of human and veterinary importance. Curr Res Parasitol Vector Borne Dis. 2022;2:100094. 35800064 10.1016/j.crpvbd.2022.100094 PMC9253710

[20] Taylor MJ, Hoerauf A, Bockarie M. Lymphatic filariasis and onchocerciasis. The Lancet. 2010;376:1175–85. 10.1016/S0140-6736(10)60586-7 20739055

[21] Cross JH. Filarial nematodes. In: Baron S, editor. Medical Microbiology. University of Texas Medical Branch at Galveston. 1996 21413271

[22] Bain O, Babayan S. Behaviour of filariae: morphological and anatomical signatures of their life style within the arthropod and vertebrate hosts. Filaria J. 2003;2:16. 14675490 10.1186/1475-2883-2-16 PMC305371

[23] Gruntmeir J, Kelly M, Ramos RAN, Verocai GG. Cutaneous filarioid nematodes of dogs in the USA: are they emerging, neglected, or underdiagnosed parasites? Front Vet Sci. 2023;10:1128611. 36908516 10.3389/fvets.2023.1128611 PMC9995907

[24] Binetruy F, Duron O. Molecular detection of *Cercopithifilaria*, *Cruorifilaria* and *Dipetalonema*-like filarial nematodes in ticks of French Guiana. Parasite. 2023;30:24. 37404115 10.1051/parasite/2023027 PMC10321233

[25] Zhang X, Norris DE, Rasgon JL. Distribution and molecular characterization of *Wolbachia* endosymbionts and filarial nematodes in Maryland populations of the lone star tick (*Amblyomma**americanum*). FEMS Microbiol Ecol. 2011;77:50–6. 21410491 10.1111/j.1574-6941.2011.01089.x PMC4118304

[26] Olmeda-Garcøa AS, Rodrøguez-Rodrøguez JA. Stage-specific development of a filarial nematode (*Dipetalonema**dracunculoides*) in vector ticks. J Helminthol. 1994;68:231–5. 7829843 10.1017/s0022149x00014395

[27] Londono MI. Behaviour and characteristics of the filarial *Dipetalonema**viteae* in the tick *Ornithodoros**tartakowskyi*. Antioquia Med. 1973;23:515–6.

[28] Bain O. Recherches sur la morphogénèse des Filaires chez l’hôte intermédiaire. Ann Parasitol Hum Comp. 1972;47:251–303.

[29] Beaver PC, Burgdorfer W. A microfilaria of exceptional size from the ixodid tick, *Ixodes**dammini*, from Shelter Island. New York J Parasitol. 1984;70:963. 6527192

[30] Untersuchungen über den Entwick- lungszyklus von *Dipetalonema rugosicauda* (syn. *Wehrdikmansia rugosicauda*) (Nematoda: Filarioidea) II. Die Entwicklung von Dipetalonema rugosicauda im Zwischenwirt *Ixodes ricinus* und Untersuchungen über das Vorkommen der Mikrofilarien im Reh (*Capreolus capreolus*). Tropenmed Parasitol. 1980;31:21–30. 7189613

[31] Yates JA, Lowrie RC. Development of *Yatesia**hydrochoerus* (Nematoda: Filarioidea) to the infective stage in Ixodid ticks. Proc Helminthol Soc Wash. 1984;51:187–90.

[32] Bezerra-Santos MA, Dantas-Torres F, Ramos RAN. *Cercopithifilaria* spp. of dogs: little known but prevalent filarioids beneath the skin. Parasit Vectors. 2023;16:386. 37880799 10.1186/s13071-023-06007-5 PMC10601261

[33] Lineberry MW, Sundstrom KD, Little SE, Allen KE. Molecular detection of *Cercopithifilaria**bainae* in brown dog ticks collected from dogs across the United States. Vet Parasitol. 2021;299:109583. 34583143 10.1016/j.vetpar.2021.109583

[34] Lineberry MW, Sundstrom KD, Little SE, Stayton EM, Allen KE. Detection of *Cercopithifilaria**bainae* infection in shelter dogs and ticks in Oklahoma, USA. Parasit Vectors. 2020;13:216. 32334624 10.1186/s13071-020-04089-z PMC7183667

[35] Bain O, Baker M, Chabaud AG. New data on the evolutionary line of *Dipetalonema* (Filarioidea. Nematoda Ann Parasitol Hum Comp. 1982;57:593–620. 6891995

[36] Brianti E, Otranto D, Dantas-Torres F, Weigl S, Latrofa MS, Gaglio G, et al. *Rhipicephalus**sanguineus* (Ixodida, Ixodidae) as intermediate host of a canine neglected filarial species with dermal microfilariae. Vet Parasitol. 2012;10:330–7. 10.1016/j.vetpar.2011.07.031 21831524

[37] Apanaskevich and Oliver. Life cycles and natural history of ticks. Oxford: Oxford University Press; 2003.

[38] Ramos RA, Giannelli A, Dantas-Torres F, Mallia E, Passantino G, Lia RP, et al. *Cercopithifilaria**rugosicauda* (Spirurida, Onchocercidae) in a roe deer and ticks from southern Italy. Int J Parasitol Parasites Wildl. 2013;2:292–6. 24533349 10.1016/j.ijppaw.2013.09.009 PMC3862540

[39] Boyd M, Santoro D, Craft WF, Ginn PE, Childress AL, Wellehan JFX, et al. Dermatitis caused by autochthonous *Cercopithifilaria**bainae* from a dog in Florida, USA: clinical, histological and parasitological diagnosis and treatment. Vet Dermatol. 2018;30:68. 30474318 10.1111/vde.12701

[40] Anderson RE. Nematode parasites of vertebrates: their development and transmission. Wallingford: CABI; 1992.

[41] Štibraniova I, Lahova M, Bartikova P. Immunomodulators in tick saliva and their benefits. Acta Virol. 2013;57:200–16. 23600877 10.4149/av_2013_02_200

[42] Šimo L, Kazimirova M, Richardson J, Bonnet SI. The essential role of tick salivary glands and saliva in tick feeding and pathogen transmission. Front Cell Infect Microbiol. 2017;7:281. 28690983 10.3389/fcimb.2017.00281 PMC5479950

[43] Moorhouse DE. The accumulation of microfilariae beneath the sites of attachment of *Ixodes**tasmani*. Trans R Soc Trop Med Hyg. 1969;63:22. 5789102

[44] Otranto D, Brianti E, Abramo F, Gaglio G, Napoli E, Latrofa MS, et al. Cutaneous distribution and localization of *Cercopithifilaria* sp. microfilariae in dogs. Vet Parasitol. 2012;190:143–50. 22698796 10.1016/j.vetpar.2012.05.016

[45] Lok J, Walker E, Glen S. Medical Entomology: A Textbook on Public Health and Veterinary Problems. 2004; Chapter. 9.

[46] Francischetti Ivo MB. The role of saliva in tick feeding. Front Biosci. 2009;14:2051–88. 10.2741/3363 PMC2785505 19273185

[47] Neelakanta G, Sultana H. Tick saliva and salivary glands: what do we know so far on their role in arthropod blood feeding and pathogen transmission. Front Cell Infect Microbiol. 2022;11:816547. 35127563 10.3389/fcimb.2021.816547 PMC8809362

[48] Uni S, Bain O, Fujita H, Matsubayashi M, Fukuda M, Takaoka H. Infective larvae of *Cercopithifilaria* spp (Nematoda: Onchocercidae) from hard ticks (Ixodidae) recovered from the Japanese serow (Bovidae). Parasite. 2013;20:1. 23340227 10.1051/parasite/2012001 PMC3718534

[49] Anteson RK. Biological studies of *Monanema**marmotae* (Webster 1967) a filarioid parasite of the woodchuck *Marmota**monax**canadensis*. Storrs, Connecticut: University of Connecticut; 1968.

[50] Böhm LK, Supperer R. Beobachtungen über eine neue Filarie (Nematoda) Wehrdikmansia *rugosicauda* Böhm & Supperer 1953, aus dem subkutanen Bindegewebe des Rehes. Berlin: Springer; 1953.

[51] Baltazard M, Chabaud AG, Minou A. Cycle evolutif d’une filaire parasite de merion. C R Hebd Seances Acad Sci. 1952;234:2115–7. 12979339

[52] Frank W. Biologie von Macdonaldius oschei (Filarioidea, Onchocercinae), zugleich ein Beitrag uber die Wirtsspezifitat von *Ornithodoros**talaje* (Ixodea) Argasidae). Zeitschrift fr Parasitenkunde. 1962;22:107–8.

[53] Bain O, Casiraghi M, Martin C, Uni S. The nematoda Filarioidea: critical analysis linking molecular and traditional approaches. Parasite. 2008;15:342–8. 18814705 10.1051/parasite/2008153342

[54] Patton TG, Dietrich G, Brandt K, Dolan MC, Piesman J, Gilmore RD. Saliva, salivary gland, and hemolymph collection from *Ixodes**scapularis* ticks. J Vis Exp. 2012;60:3894. 10.3791/3894 PMC3912584 22371172

[55] Santos MAB, de Macedo LO, Otranto D, Ramos CADN, Rêgo AGOD, Giannelli A, et al. Screening of *Cercopithifilaria**bainae* and *Hepatozoon**canis* in ticks collected from dogs of Northeastern Brazil. Acta Parasitol. 2018;63:605–8. 29975651 10.1515/ap-2018-0069

[56] Spratt DM, Haycock P. Aspects of the life history of *Cercopithifilaria**johnstoni* (Nematoda:Filarioidea). Int J Parasitol. 1988;18:1087–92. 3220649 10.1016/0020-7519(88)90079-3

[57] Petit G, Bain O, Carrat C, Marval F. Development of the filaria *Monanema**martini* in the epidermis of ixodid ticks. Ann Parasitol Hum Comp. 1988;63:54–63. 3400961 10.1051/parasite/198863154

[58] Ebmer D, Handschuh S, Schwaha T, Rubio-García A, Gärtner U, Glösmann M, et al. Novel 3D in situ visualization of seal heartworm (*Acanthocheilonema**spirocauda*) larvae in the seal louse (*Echinophthirius**horridus*) by X-ray microCT. Sci Rep. 2022;12:14181. 35982240 10.1038/s41598-022-18418-y PMC9388652

[59] O’Sullivan JDB, Behnsen J, Starborg T, MacDonald AS, Phythian-Adams AT, Else KJ, et al. X-ray micro-computed tomography (μCT): an emerging opportunity in parasite imaging. Parasitology. 2018;145:848–54. 29179788 10.1017/S0031182017002074 PMC6088774

[60] Leidenberger S, Harding K, Härkönen T. Phocid seals, seal lice and heartworms: a terrestrial host-parasite system conveyed to the marine environment. Dis Aquat Organ. 2007;77:235–53. 18062474 10.3354/dao01823

[61] Hall MJR, Martin-Vega D, Clark B, Ghosh D, Rogers M, Pigoli D, et al. Micro-CT imaging of Onchocerca infection of *Simulium**damnosum* s.l. blackflies and comparison of the peritrophic membrane thickness of forest and Savannah flies. Med Vet Entomol. 2021;35:231–8. 33480060 10.1111/mve.12509 PMC8451916

[62] Latrofa MS, Angelou A, Giannelli A, Annoscia G, Ravagnan S, Dantas-Torres F, et al. Ticks and associated pathogens in dogs from Greece. Parasit Vectors. 2017;10:301. 28645329 10.1186/s13071-017-2225-2 PMC5481936

[63] Latrofa MS, Dantas-Torres F, Giannelli A, Otranto D. Molecular detection of tick-borne pathogens in *Rhipicephalus**sanguineus* group ticks. Ticks Tick Borne Dis. 2014;5:943–6. 25113982 10.1016/j.ttbdis.2014.07.014

[64] Santos MAB, de Souza IB, de Macedo LO, et al. *Cercopithifilaria**bainae* in *Rhipicephalus**sanguineus* sensu lato ticks from dogs in Brazil. Ticks Tick Borne Dis. 2017;8:623–5. 28442240 10.1016/j.ttbdis.2017.04.007

[65] Otranto D, Brianti E, Dantas-Torres F, Weigl S, Latrofa MS, Gaglio G, et al. Morphological and molecular data on the dermal microfilariae of a species of *Cercopithifilaria* from a dog in Sicily. Vet Parasitol. 2011;182:221–9. 21705146 10.1016/j.vetpar.2011.05.043

[66] Tokarz R, Tagliafierro T, Ian Lipkin W, Marques AR. Characterization of a *Monanema* nematode in *Ixodes**scapularis*. Parasit Vectors. 2020;13:364. 32709241 10.1186/s13071-020-04228-6 PMC7379800

[67] Henning TC, Orr JM, Smith JD, Arias JR, Rasgon JL, Norris DE. Discovery of filarial nematode DNA in *Amblyomma**americanum* in Northern Virginia. Ticks Tick Borne Dis. 2016;7:315–8. 26707835 10.1016/j.ttbdis.2015.11.007 PMC4876860

[68] Bruley M, Duron O. Multi-locus sequence analysis unveils a novel genus of filarial nematodes associated with ticks in French Guiana. Parasite. 2024;31:14. 38488705 10.1051/parasite/2024015 PMC10941835

[69] Ramos RAN, Giannelli A, Carbone D, Baneth G, Dantas-Torres F, Otranto D. Occurrence of *Hepatozoon**canis* and *Cercopithifilaria**bainae* in an off-host population of *Rhipicephalus**sanguineus* sensu lato ticks. Ticks Tick Borne Dis. 2014;5:311–4. 24594107 10.1016/j.ttbdis.2013.12.005

[70] Baneth G. Tick-borne infections of animals and humans: a common ground. Int J Parasitol. 2014;44:591–6. 24846527 10.1016/j.ijpara.2014.03.011

[71] Chicana B, Couper LI, Kwan JY, Tahiraj E, Swei A. Comparative microbiome profiles of sympatric tick species from the far-western United States. Insects. 2019;10:353. 31635285 10.3390/insects10100353 PMC6836157

[72] Greay TL, Gofton AW, Paparini A, Ryan UM, Oskam CL, Irwin PJ. Recent insights into the tick microbiome gained through next-generation sequencing. Parasit Vectors. 2018;11:12. 29301588 10.1186/s13071-017-2550-5 PMC5755153

[73] Moutailler S, Valiente Moro C, Vaumourin E, Michelet L, Tran FH, Devillers E, et al. Co-infection of ticks: the rule rather than the exception. PLoS Negl Trop Dis. 2016;10:e0004539. 26986203 10.1371/journal.pntd.0004539 PMC4795628

[74] Aliota MT, Chen CC, Dagoro H, Fuchs JF, Christensen BM. Filarial worms reduce plasmodium infectivity in mosquitoes. PLoS Negl Trop Dis. 2011;5:e963. 21347449 10.1371/journal.pntd.0000963 PMC3035669

[75] Mellor PS, Boorman J. Multiplication of bluetongue virus in *culicoides**nubeculosus* (Meigen) simultaneously infected with the virus and the microfilariae of *Onchocerca**cervicalis* (Railliet & Henry). Ann Trop Med Parasitol. 1980;74:463–9. 6257192 10.1080/00034983.1980.11687368

[76] Eisen RJ, Kugeler KJ, Eisen L, Beard CB, Paddock CD. Tick-borne Zoonoses in the United States: persistent and emerging threats to human health. ILAR J. 2017;58:319–35. 28369515 10.1093/ilar/ilx005 PMC5610605

[77] Sonenshine DE. Range expansion of tick disease vectors in North America: implications for spread of tick-borne disease. Int J Environ Res Public Health. 2018;15:478. 29522469 10.3390/ijerph15030478 PMC5877023

[78] Van Treuren W, Ponnusamy L, Brinkerhoff RJ, Gonzalez A, Parobek CM, Juliano JJ, et al. Variation in the microbiota of *Ixodes* ticks with regard to geography, species, and sex. Appl Environ Microbiol. 2015;81:6200–9. 26150449 10.1128/AEM.01562-15 PMC4542252

[79] Qiu Y, Nakao R, Ohnuma A, Kawamori F, Sugimoto C. Microbial population analysis of the salivary glands of ticks; a possible strategy for the surveillance of bacterial pathogens. PLoS ONE. 2014;9:e103961. 25089898 10.1371/journal.pone.0103961 PMC4121176

[80] Couper LI, Kwan JY, Ma J, Swei A. Drivers and patterns of microbial community assembly in a Lyme disease vector. Ecol Evol. 2019;9:7768–79. 31346439 10.1002/ece3.5361 PMC6635933

[81] Swei A, Kwan JY. Tick microbiome and pathogen acquisition altered by host blood meal. ISME J. 2016;11:813–6. 27858931 10.1038/ismej.2016.152 PMC5322304

[82] Gall CA, Reif KE, Scoles GA, Mason KL, Mousel M, Noh SM, et al. The bacterial microbiome of *Dermacentor**andersoni* ticks influences pathogen susceptibility. ISME J. 2016;10:1846–55. 26882265 10.1038/ismej.2015.266 PMC5029153

[83] Pampiglione S, Canestri Trotti G, Marchetti S. Ritrovamento di *Diptalonema**grassii* (Noè, 1907) in *Rhipicephalus**sanguineus* su cane in Italia e descrizione di alcuni suoi stadi larvali. Parassitologia. 1983;25:316.

[84] Bianco AE, Muller R, Nelson GS. Biology of *Monanema**globulosa*, a rodent filaria with skin-dwelling microfilariae. J Helminthol. 1983;57:259–78.

[85] Londono IM. Transmission of microfilariae and infective larvae of *Dipetalonema**viteae* (Filarioidea) among vector ticks, *Ornithodoros**tartakowskyi* (Argasidae), and loss of microfilariae in coxal fluid. J Parasitology. 1976;62:786–8. 988155

[86] Bain O, Chabaud A, Duke BOL, Kouznetzov R, Mullet RL, Wenk P. Litosoma Wite Krepkogorskaya, 1933 (Nematoda), Proposed correction to Litosoma viteae Z.n.(S) 2203. Bull Zool Nomencl. 1978;35:51–4.

[87] Bain O, Petit G, Jacquet-viallet P, Houin R. *Cherylla guyanensis* n. gen., n. sp., – Filaire d’un Marsupial sud-américain, transmise par tique. Ann Parasitol Hum Comp. 1985;60:727–37.

[88] Vinnie-Siow WY, Low VL, Tan TK, Wong ML, Leong CS, Ahmad NW, et al. Identification of potential vectors of *Dirofilaria immitis* and *Brugia pahangi* (Spirurida: Filariidae): first observation of infective third-stage larva of *B pahangi* in *Culex quinquefasciatus* (Diptera: Culicidae). Pathog Glob Health. 2022;116:356–64. 35287548 10.1080/20477724.2022.2035624 PMC9387329

[89] Ledesma N, Harrington L. Mosquito vectors of dog heartworm in the United States: vector status and factors influencing transmission efficiency. Top Companion Anim Med. 2011;26:178–85. 22152605 10.1053/j.tcam.2011.09.005

[90] Smith AA, Pal U. Immunity-related genes in *Ixodes**scapularis* perspectives from genome information. Front Cell Infect Microbiol. 2014;4:116. 25202684 10.3389/fcimb.2014.00116 PMC4141456

[91] Hajdušek O, Šíma R, Ayllón N, Jalovecká M, Perner J, de la Fuente J, et al. Interaction of the tick immune system with transmitted pathogens. Front Cell Infect Microbiol. 2013;3:26. 23875177 10.3389/fcimb.2013.00026 PMC3712896

[92] Narasimhan S, Swei A, Abouneameh S, Pal U, Pedra JHF, Fikrig E. Grappling with the tick microbiome. Trends Parasitol. 2021;37:722–33. 33962878 10.1016/j.pt.2021.04.004 PMC8282638

[93] Uni S, Bain O, Suzuki K, Agatsuma T, Harada M, Motokawa M, et al. *Acanthocheilonema delicata* n. sp. (Nematoda: Filarioidea) from Japanese badgers (Meles anakuma): description, molecular identification, and *Wolbachia* screening. Parasitol Int. 2013;62:14–23. 22926421 10.1016/j.parint.2012.08.004

[94] Vicente JJ, Rodrigues HO, Gomes DC, Pinto RM. Nematóides do Brasil. Parte V: Nematóides de mamíferos. Rev Bras Zool. 1997;14:1–452.

[95] Eberhard ML, Morales GA, Orihel TC. *Cruorifilaria tuberocauda * gen. et sp. n. (Nematoda: Filarioidea) from the Capybara *Hydrochoerus hydrochaeris* in Colombia J Parasitol. 1976;62:604. 957038

[96] Morales GA, Guzman VH, Angel D. Vascular damage is caused by *Cruorifilaria**tuberocauda* in the capybara (*Hydrochoerus**hydrochaeris*). J Wildl Dis. 1978;14:15–21. 633511 10.7589/0090-3558-14.1.15

[97] Spratt DM. *Monanema australe* sp. nov (Nematoda: Onchocercidae) from the lungs and liver of native rodents in North Queensland. Trans R Soc S Aust. 2008;132:1–6.

[98] Webster WA. *Ackertia marmotae* n. sp. (Filarioidea: Onchocercinae) from the groundhog (*Marmota monax*). Can J Zool. 1967;45:277–83.

[99] Junker K, Medger K, Lutermann H, Bain O. *Monanema joopin*. Sp. (Nematoda, Onchocercidae) from *Acomys* (Acomys) *spinosissimus* Peters, 1852 (Muridae) in South Africa, with comments on the filarial genus. Parasite. 2012;19:331–40. 23193517 10.1051/parasite/2012194331 PMC3671456

[100] Bain O, Bartlett C, Petit G. Une Filaire de Muridés africains dans la paroi du colon, *Monanema martinin* sp. Ann Parasitol Hum Comp. 1986;61:465–72. 3813428 10.1051/parasite/1986614465

[101] Yates JA, Jorgenson JP. *Dipetalonema* (Alafilaria) *hydrochoerus* subgen. et sp. N. (Nematoda: Filarioidea) from Colombian Capybaras. J Parasitol. 1983;69:606. 6685177

[102] Andersson MO, Chitimia-Dobler L. Detection of *Cercopithifilaria**bainae* in western Romania. Parasitol Res. 2017;116:3235–8. 28956159 10.1007/s00436-017-5625-5

[103] Angelou A, Latrofa MS, Annoscia G, Symeonidou I, Theodoridis A, Polizopoulou ZS, et al. *Cercopithifilaria* species in dogs and ticks from Greece. Parasitol Res. 2020;119:3391–400. 32607708 10.1007/s00436-020-06784-3

[104] Olmeda-Garcøa AS, Rodrøguez-Rodrøguez JA. Experimental transmission of *Dipetalonema**dracunculoides* (Cobbold 1870) by *Rhipicephalus**sanguineus* (Latreille 1806). Vet Parasit. 1993;27:339–42. 10.1016/0304-4017(93)90034-k 8333138

[105] Singh DP, Chatterjee RK, Sen AB. Studies on susceptibility of *Ornithodoros**moubata* as vector of *Dipetalonema**viteae* (Filariodea). J Commun Dis. 1988;20:220–5. 3256567

[106] Otranto D, Brianti E, Dantas-Torres F, Miró G, Latrofa MS, Mutafchiev Y, et al. Species diversity of dermal microfilariae of the genus *Cercopithifilaria* infesting dogs in the Mediterranean region. Parasitology. 2013;140:99–108. 22914299 10.1017/S0031182012001357

[107] Bain O, Petit G, Gueye A. Transmission expérimentale de *Monanema**nilotica* El Bihari et coll., 1977, filaire à microfilaires dermiques parasite de Muridés africains [Experimental transmission of *Monanema**nilotica* El Bihari & coll., 1977, a filaria with skin-dwelling microfilaria parasitic in African murids]. Ann Parasitol Hum Comp. 1985;60:83–9. 3985535 10.1051/parasite/198560183

[108] Dantas-Torres F, de Sousa-Paula LC, Otranto D. The *Rhipicephalus sanguineus* group updated list of species geographical distribution, and vector competence. Parasit Vectors. 2024;17:540. 39731169 10.1186/s13071-024-06572-3 PMC11681662

